# Supplementary material for: ADAR1 haploinsufficiency and sustained picornaviral RdRp dsRNA synthesis synergize to dysregulate RNA editing and cause multi-system interferonopathy
Source: mBio. 2025 Jul 22;16(8):e01492-25. doi: 10.1128/mbio.01492-25 (PMC12345148; doi:10.1128/mbio.01492-25)
Supplement: Supplemental Material — Fig. S1–S10; supplemental methods. [file mbio.01492-25-s0001.pdf]

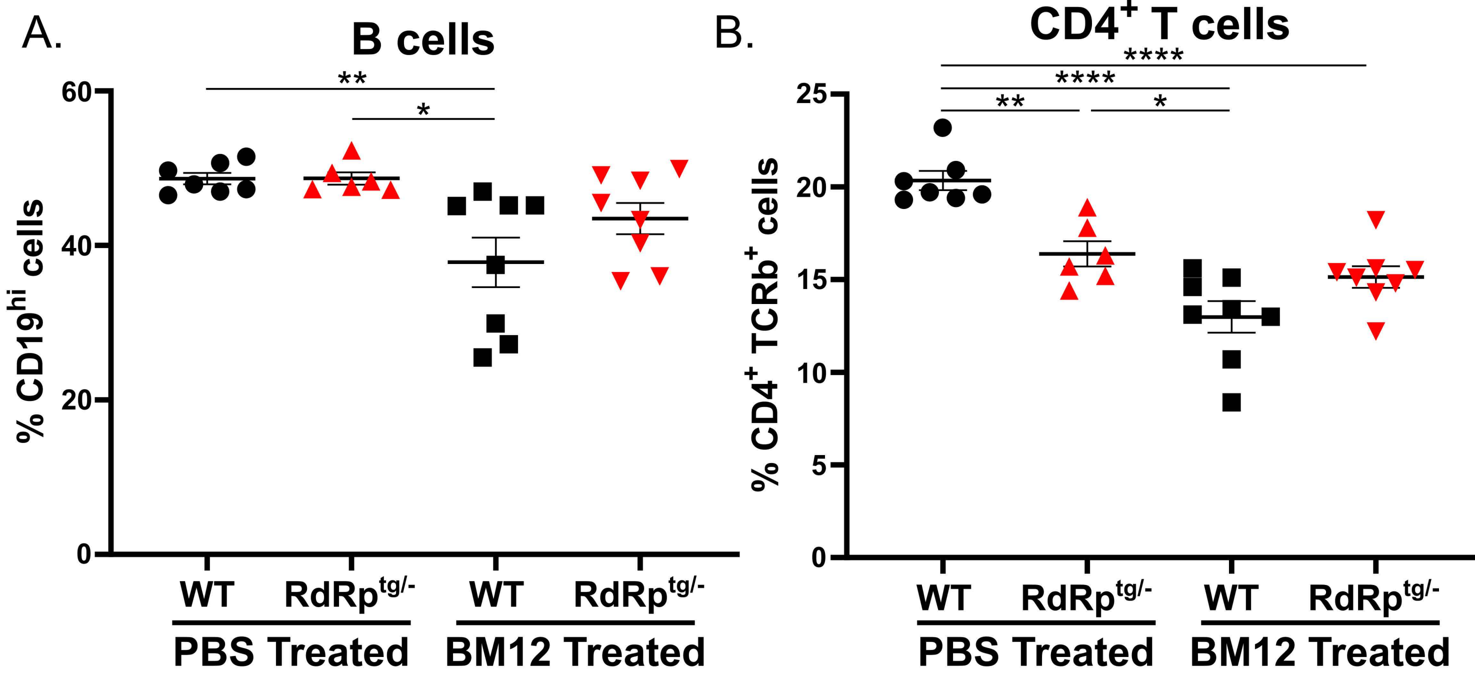


Supplemental Figure 1

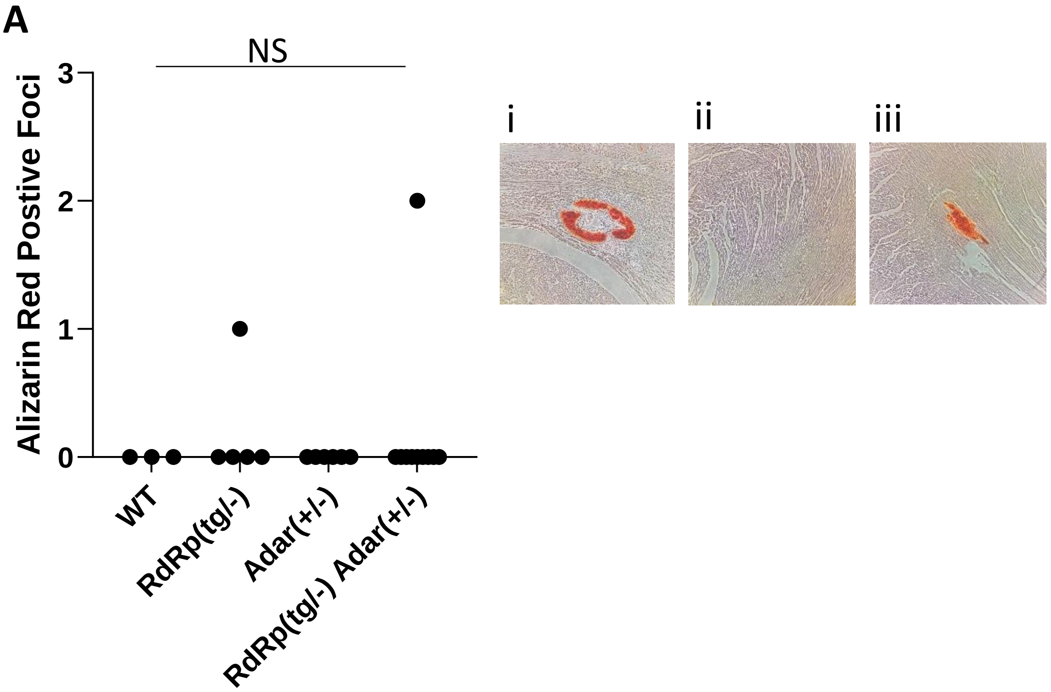


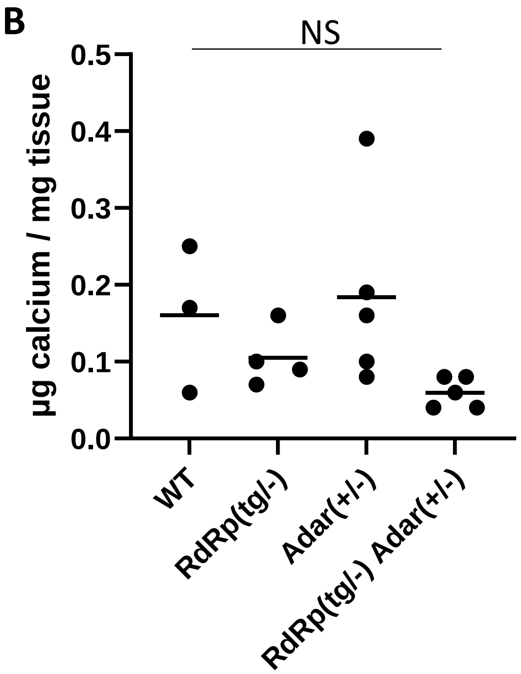


Supplemental Figure 2

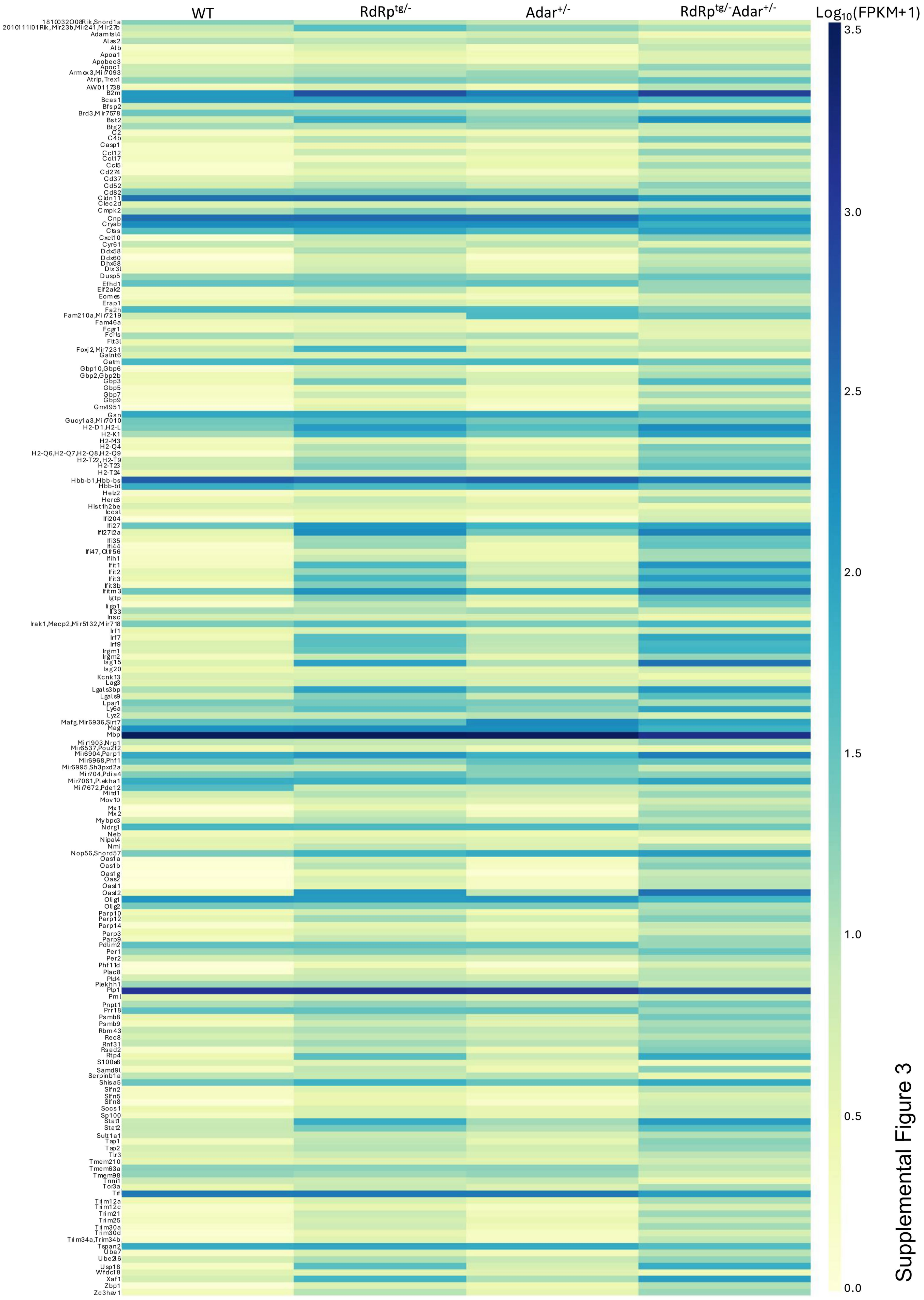


Supplemental Figure 3

| | WT vs. RdRP | | WT vs. ADAR | | WT vs. ADAR-RdRP | | RdRP vs. ADAR | | RdRP vs. ADAR-RdRP | | ADAR vs. ADAR-RdRP | |
|---|---|---|---|---|---|---|---|---|---|---|---|---|
| **Diseases or Functions Annotation** | **-log(B-H p-value)** | **z-score** | **-log(B-H p-value)** | **z-score** | **-log(B-H p-value)** | **z-score** | **-log(B-H p-value)** | **z-score** | **-log(B-H p-value)** | **z-score** | **-log(B-H p-value)** | **z-score** |
| Antimicrobial response | 0.00000 | **2.39** | 1.97E-09 | 1.455 | NaN | NaN | 0.00000 | **-2.39** | 1.08E-05 | NaN | 1.21E-12 | 0.995 |
| Antiviral response | 0.00000 | **2.39** | 2.77E-11 | 1.455 | NaN | NaN | 0.00000 | **-2.39** | 2.82E-06 | NaN | 6.75E-12 | 0.995 |
| Apoptosis of breast cancer cell lines | NaN | NaN | NaN | NaN | NaN | NaN | NaN | NaN | NaN | NaN | 0.00146 | **2.808** |
| Apoptosis of epithelial cells | NaN | NaN | NaN | NaN | NaN | NaN | NaN | NaN | NaN | NaN | 0.00003 | **2.55** |
| Apoptosis of liver cells | NaN | NaN | NaN | NaN | NaN | NaN | NaN | NaN | NaN | NaN | 0.00146 | **2.169** |
| Cell death of epithelial cells | 0.00251 | **2.414** | NaN | NaN | NaN | NaN | NaN | NaN | 1.06E-03 | 0.922 | 1.41E-05 | **3.058** |
| Demyelination of nerves | NaN | NaN | NaN | NaN | NaN | NaN | NaN | NaN | 0.00001 | **2.195** | NaN | NaN |
| Differentiation of Th17 cells | NaN | NaN | NaN | NaN | NaN | NaN | | | NaN | NaN | 0.00014 | **-2.382** |
| Encephalitis | 3.09E-04 | -1.925 | 3.72E-07 | 0.264 | NaN | NaN | NaN | NaN | 1.09E-04 | -1.361 | 0.00000 | **-3.488** |
| Experimental autoimmune encephalomyelitis | 5.09E-04 | -1.639 | 7.57E-07 | 0.659 | NaN | NaN | 1.45E-06 | 1.881 | 1.66E-04 | -1.37 | 0.00000 | **-3.326** |
| Inflammation of absolute anatomical region | 8.46E-06 | -1.905 | 5.51E-08 | -0.527 | NaN | NaN | 1.39E-07 | 1.365 | 0.00002 | **-2.537** | 0.00000 | **-3.351** |
| Inflammation of organ | 3.08E-05 | -1.264 | 1.75E-07 | 0.255 | NaN | NaN | 1.71E-07 | 1.078 | 0.00001 | **-2.346** | 0.00000 | **-2.881** |
| Inhibition of cells | NaN | NaN | NaN | NaN | NaN | NaN | NaN | NaN | NaN | NaN | 0.00142 | **2.166** |
| Maturation of phagocytes | NaN | NaN | 2.29E-03 | -0.169 | NaN | NaN | 0.00074 | **-2.183** | NaN | NaN | 5.47E-04 | 0.891 |
| Necrosis | 8.48E-03 | 1.117 | NaN | NaN | NaN | NaN | NaN | NaN | 0.00160 | **2.156** | 0.00004 | **2.291** |
| Necrosis of epithelial tissue | NaN | NaN | NaN | NaN | NaN | NaN | NaN | NaN | 1.36E-04 | 1.014 | 0.00000 | **2.072** |
| Neuronal cell death | NaN | NaN | NaN | NaN | NaN | NaN | NaN | NaN | 0.00476 | **2.302** | 0.00006 | **2.309** |
| Proliferation of antigen presenting cells | NaN | NaN | NaN | NaN | NaN | NaN | 2.26E-03 | -0.865 | 3.64E-03 | NaN | 0.00014 | **2.201** |
| Proliferation of blood cells | 0.00421 | **2.084** | NaN | NaN | NaN | NaN | NaN | NaN | 9.98E-04 | 0.317 | 8.85E-06 | 0.535 |
| Proliferation of immune cells | 0.00429 | **2.084** | NaN | NaN | NaN | NaN | NaN | NaN | 9.14E-04 | 0.358 | 1.70E-05 | 0.808 |
| Quantity of blood cells | 0.00064 | **2.383** | NaN | NaN | NaN | NaN | 0.00193 | **-2.021** | 1.63E-04 | 0.487 | 2.79E-06 | 0.67 |
| Quantity of IL-12 in blood | 0.00002 | **-2** | 6.62E-03 | NaN | NaN | NaN | 0.00002 | **2** | NaN | NaN | 0.00007 | **-2** |
| Quantity of interleukin | NaN | NaN | NaN | NaN | NaN | NaN | NaN | NaN | NaN | NaN | 0.00064 | **-2** |
| Quantity of leukocytes | 0.00046 | **2.295** | NaN | NaN | NaN | NaN | 1.60E-03 | -1.906 | 1.02E-04 | 0.167 | 9.96E-07 | 0.336 |
| Quantity of phagocytes | 0.00104 | **2.191** | NaN | NaN | NaN | NaN | 2.21E-03 | -1.544 | 2.25E-05 | -0.125 | 5.80E-05 | -0.102 |
| Quantity of protein in blood | 0.00769 | **-2.909** | NaN | NaN | NaN | NaN | 0.00034 | **2.088** | NaN | NaN | 6.48E-04 | -1.717 |
| Rheumatic Disease | 0.00000 | **-2.219** | 7.94E-05 | -1 | NaN | NaN | 7.74E-07 | 1.179 | 3.07E-05 | -0.503 | 2.99E-08 | -1.157 |

Supplemental Figure 4

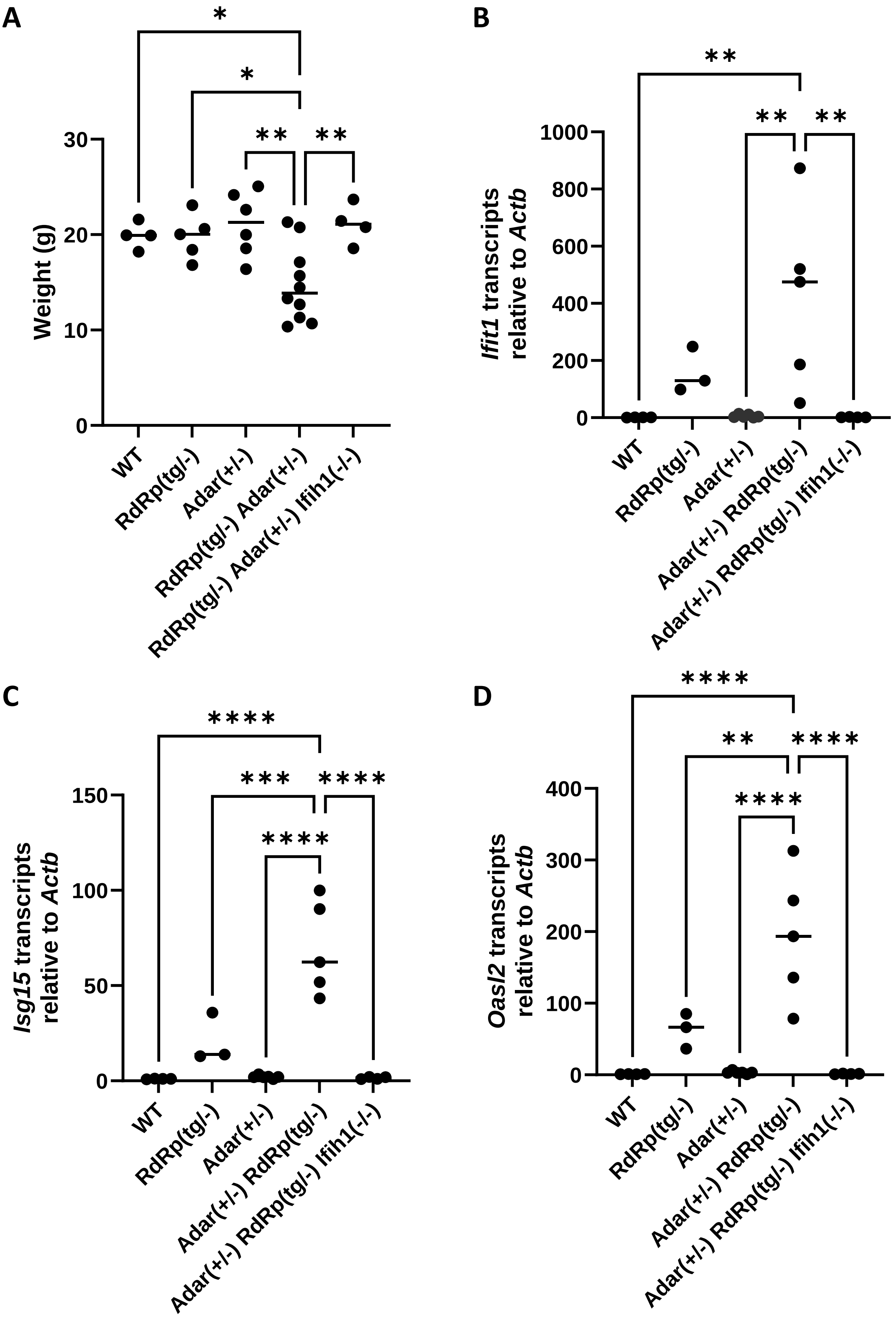


Supplemental Figure 5

| Diseases or Functions Annotation | WT vs. RdRp$^{tg/-}$ | WT vs. Adar$^{+/-}$ | WT vs. RdRp$^{tg/-}$ Adar$^{+/-}$ | RdRp$^{tg/-}$ vs. Adar$^{+/-}$ | RdRp$^{tg/-}$ vs. RdRp$^{tg/-}$ Adar$^{+/-}$ | Adar$^{+/-}$ vs. RdRp$^{tg/-}$ Adar$^{+/-}$ |
|---|---|---|---|---|---|---|
| Activation of cells | 2.339 | 1.88 | 2.069 | -2.262 | 1.501 | 2.154 |
| Activation of macrophages | 1.803 | 1.282 | 2.145 | -1.803 | 1.898 | 1.633 |
| Activation of myeloid cells | 1.472 | 0.943 | 2.084 | -1.472 | 2.095 | 1.698 |
| Activation of phagocytes | 1.469 | 0.938 | 1.765 | -1.469 | 2.095 | 1.795 |
| Adhesion of blood cells | 1.989 | NA | 2.637 | NA | 2.132 | 2.025 |
| Antigen presentation | 2.414 | NA | 2.414 | NA | NA | 2.414 |
| Antigen presentation by cells | 2 | NA | 2 | NA | NA | 2 |
| Antimicrobial response | 2.787 | 2.406 | 2.792 | -2.947 | NA | 2.792 |
| Antiviral response | 2.787 | 2.406 | 2.792 | -2.947 | NA | 2.792 |
| Antiviral response of cells | 2.219 | 1.982 | 1.31 | -2.219 | NA | 1.31 |
| Apoptosis | 0.916 | 0.197 | 1.616 | -0.696 | 1.157 | 2.443 |
| Apoptosis of tumor cell lines | NA | NA | 1.173 | -0.889 | NA | 2.09 |
| Autophagy of cells | 2.072 | NA | 1.363 | -1.502 | 1.095 | 1.152 |
| Cell cycle progression | 2.961 | NA | 2.195 | -2.809 | NA | 2.448 |
| Cell death of epithelial cells | NA | NA | 1.966 | NA | NA | 2.68 |
| Cell death of tumor cell lines | 1.044 | NA | 1.396 | -1.388 | NA | 2.158 |
| Cell movement of leukocytes | 2.904 | NA | 1.35 | -2.394 | 1.501 | 1.387 |
| Cell movement of monocytes | 2.185 | NA | 2.083 | NA | 1.571 | 2.083 |
| Cell movement of T lymphocytes | 2.544 | 1.948 | 1.072 | -1.824 | 1.645 | 1.072 |
| Cellular homeostasis | 1.314 | 1.026 | 2.089 | -1.662 | 0.957 | 1.356 |
| Cellular infiltration | 2.619 | NA | 0.842 | -1.692 | 1.402 | 1.042 |
| Cellular infiltration by leukocytes | 2.696 | 2.342 | 1.081 | -2.13 | 1.481 | 1.294 |
| Cellular infiltration by lymphocytes | 2.187 | NA | 0.201 | -1.507 | 0.692 | 0.201 |
| Cellular infiltration by myeloid cells | 1.878 | 2.091 | 0.83 | NA | 0.935 | 0.791 |
| Contact growth inhibition | 2.232 | NA | 2.467 | -2.052 | NA | 2.467 |
| Contact growth inhibition of tumor | NA | NA | 2.022 | -1.49 | NA | 2.022 |
| Coordination | NA | NA | NA | NA | -2.401 | NA |
| Cytostasis | 2.442 | NA | 2.742 | -2.522 | NA | 2.742 |
| Cytostasis of tumor cell lines | 1.972 | 1.451 | NA | -1.762 | NA | 2.232 |
| Cytotoxicity of cytotoxic T cells | 2.439 | NA | 2.635 | -1.988 | NA | 2.438 |
| Cytotoxicity of lymphocytes | 2.794 | NA | 2.605 | -2.048 | NA | 2.412 |
| Cytotoxicity of T lymphocytes | NA | NA | 2.332 | -1.583 | NA | 2.112 |
| Degradation of DNA | NA | NA | 2.781 | NA | NA | 2.781 |
| Demyelination of nerves | NA | NA | 1.798 | NA | 2.057 | 1.541 |
| Differentiation of T lymphocytes | 1.859 | NA | 1.227 | -2.1 | NA | 0.815 |
| Elimination of cells | NA | NA | 2.2 | NA | 1.964 | 2.2 |
| Encephalitis | -1.515 | -1 | -2.141 | NA | -1.405 | -2.141 |
| Endocytosis | 2.6 | NA | NA | NA | NA | 0.566 |
| Fragmentation of DNA | NA | NA | 2.598 | NA | NA | 2.598 |
| Hematologic cancer | NA | NA | -2.752 | NA | NA | -2.752 |
| Hematologic cancer of cells | NA | NA | -2.396 | NA | NA | -2.396 |
| Hematopoietic neoplasm | NA | NA | -2.032 | NA | NA | -2.032 |
| Immune response of brain | -1.745 | NA | -2.311 | 1.992 | NA | -2.311 |
| Immune response of cells | 3.924 | 3.1 | 2.981 | -3.066 | NA | 2.445 |
| Immune response of leukocytes | 2.415 | 1.981 | 0.755 | -1.218 | 1.004 | 0.08 |
| Infection by Flaviviridae | -1.732 | -1.732 | -2 | 1.732 | NA | -2 |
| Infection by Herpesviridae | NA | NA | -2.187 | NA | NA | -2.187 |
| Infection by RNA virus | -1.879 | -2.019 | -2.719 | 2.088 | -1.388 | -2.745 |
| Infection of cells | -1.152 | NA | -2.341 | 1.417 | -1.2 | -2.371 |
| Infection of kidney cell lines | -1.651 | -1.337 | -2.093 | 1.651 | NA | -2.291 |
| Infection of mammalia | -3.48 | -2.662 | -3.452 | 3.388 | -1.445 | -3.207 |
| Infiltration by T lymphocytes | 2.187 | NA | 0.052 | -1.507 | 0.842 | 0.052 |
| Inflammation of absolute anatomic | -1.386 | -0.851 | -2.132 | 1.358 | -0.958 | -2.132 |
| Inflammation of organ | -1.311 | -0.729 | -2.222 | 1.29 | -0.76 | -2.173 |
| Inhibition of lymphocytes | NA | NA | 2.172 | NA | NA | 2.172 |
| Inhibition of RNA virus | 1.974 | NA | 2.196 | -1.974 | NA | 1.568 |
| Inhibition of virus | NA | NA | 2.415 | -2.211 | NA | 1.848 |
| Innate immune response | 2.63 | 2.219 | 3.43 | -3.132 | 2.8 | 3.43 |
| Leukocyte migration | 2.991 | NA | 1.565 | -2.488 | 1.505 | 1.326 |
| Lymphatic system tumor | NA | NA | -2.592 | NA | NA | -2.592 |
| Lymphocyte migration | 2.721 | 2.144 | 0.753 | -2.044 | 1.211 | 0.753 |
| Lymphocytic cancer | NA | NA | -2.593 | NA | NA | -2.593 |
| Lymphocytic neoplasm | NA | NA | -2.593 | NA | NA | -2.593 |
| Lymphohematopoietic cancer | NA | NA | -2.752 | NA | NA | -2.752 |
| Lymphohematopoietic neoplasia | NA | NA | -2.032 | NA | NA | -2.032 |
| Lymphoid cancer | NA | NA | -2.593 | NA | NA | -2.593 |
| Lymphoreticular neoplasm | NA | NA | -2.4 | NA | NA | -2.4 |
| Migration of cancer cells | 1.342 | 2 | 0.213 | -1.342 | 0.956 | 0.508 |
| Migration of cells | 3.179 | 2.898 | 1.999 | -2.573 | 0.825 | 1.285 |
| Morbidity or mortality | -2.789 | NA | -1.199 | NA | 0.575 | -0.651 |
| Necrosis | 1.494 | 1.476 | 2.39 | -1.714 | 2.274 | 2.989 |
| Necrosis of epithelial tissue | NA | NA | 1.737 | NA | NA | 2.346 |
| Neoplasia of blood cells | NA | NA | -2.512 | NA | NA | -2.512 |
| Neoplasia of leukocytes | NA | NA | -2.396 | NA | NA | -2.396 |
| Neuromuscular disease | 2.219 | 1.982 | 1.664 | -1.982 | 1.387 | 1.664 |
| Polarization of blood cells | NA | NA | NA | NA | 2 | NA |
| Polarization of cells | NA | 1.982 | NA | NA | 2.219 | NA |
| Production of protein | 1.53 | 1.217 | 1.148 | -2.096 | 0.485 | 0.678 |
| Quantity of blood cells | 2.217 | 1.36 | 0.079 | -2.117 | 0.041 | 0.653 |
| Quantity of CD8+ T lymphocyte | 2.388 | 2.191 | 1.721 | -2.207 | 1.461 | 1.721 |
| Quantity of cytokine | NA | NA | -2.343 | NA | NA | -2.154 |
| Quantity of IFNG in blood | -2.183 | NA | -2.4 | 2.183 | NA | -2.4 |
| Quantity of MHC Class I on cell surf | 2.207 | NA | 2.207 | -1.981 | NA | 2.207 |
| Recruitment of leukocytes | 2.339 | 2.339 | 2.156 | -1.666 | 1.812 | 1.089 |
| Recruitment of T lymphocytes | 1.96 | 1.96 | 2.199 | -1.96 | 1.996 | NA |
| Replication of Flaviviridae | -2.643 | -2.643 | -2.988 | 2.826 | -2.275 | -2.988 |
| Replication of flavivirus | -1.963 | -2.181 | -2.181 | 2.181 | -1.939 | -2.181 |
| Replication of Hepatitis C virus | -2.335 | -2.111 | -2.528 | 2.324 | NA | -2.528 |
| Replication of Herpesviridae | -2.132 | -2.828 | -3.175 | 2.525 | -2.236 | -3.175 |
| Replication of Murine herpesvirus 4 | -2.236 | -2 | -2.828 | 2.236 | NA | -2.828 |
| Replication of RNA virus | -2.875 | -3.077 | -3.617 | 3.292 | -2.304 | -3.497 |
| Replication of vesicular stomatitis | -2.679 | -2.473 | -3.129 | 2.841 | -1.702 | -3.129 |
| Replication of viral replicon | -2.818 | -3.115 | -3.56 | 3.271 | -2.782 | -3.56 |
| Replication of virus | -3.082 | -3.443 | -3.924 | 3.583 | -2.607 | -3.812 |
| Response of antigen presenting cel | 2.219 | NA | 1.65 | NA | NA | 0.799 |
| Response of embryonic cell lines | 1.96 | NA | 2.4 | -2.2 | NA | 2.4 |
| Response of epithelial cell lines | 1.969 | NA | 2.404 | -2.207 | NA | 2.404 |
| Response of kidney cell lines | 1.969 | NA | 2.404 | -2.207 | NA | 2.404 |
| Response of phagocytes | 2.207 | 2.207 | NA | -2.207 | NA | 0.845 |
| Transactivation of RNA | NA | 1.961 | NA | -2.41 | NA | NA |
| Viral Infection | -3.615 | -3.851 | -4.1 | 3.801 | -2.583 | -3.943 |
| Viral life cycle | NA | NA | -3.683 | NA | NA | -3.683 |

Supplemental Figure 6

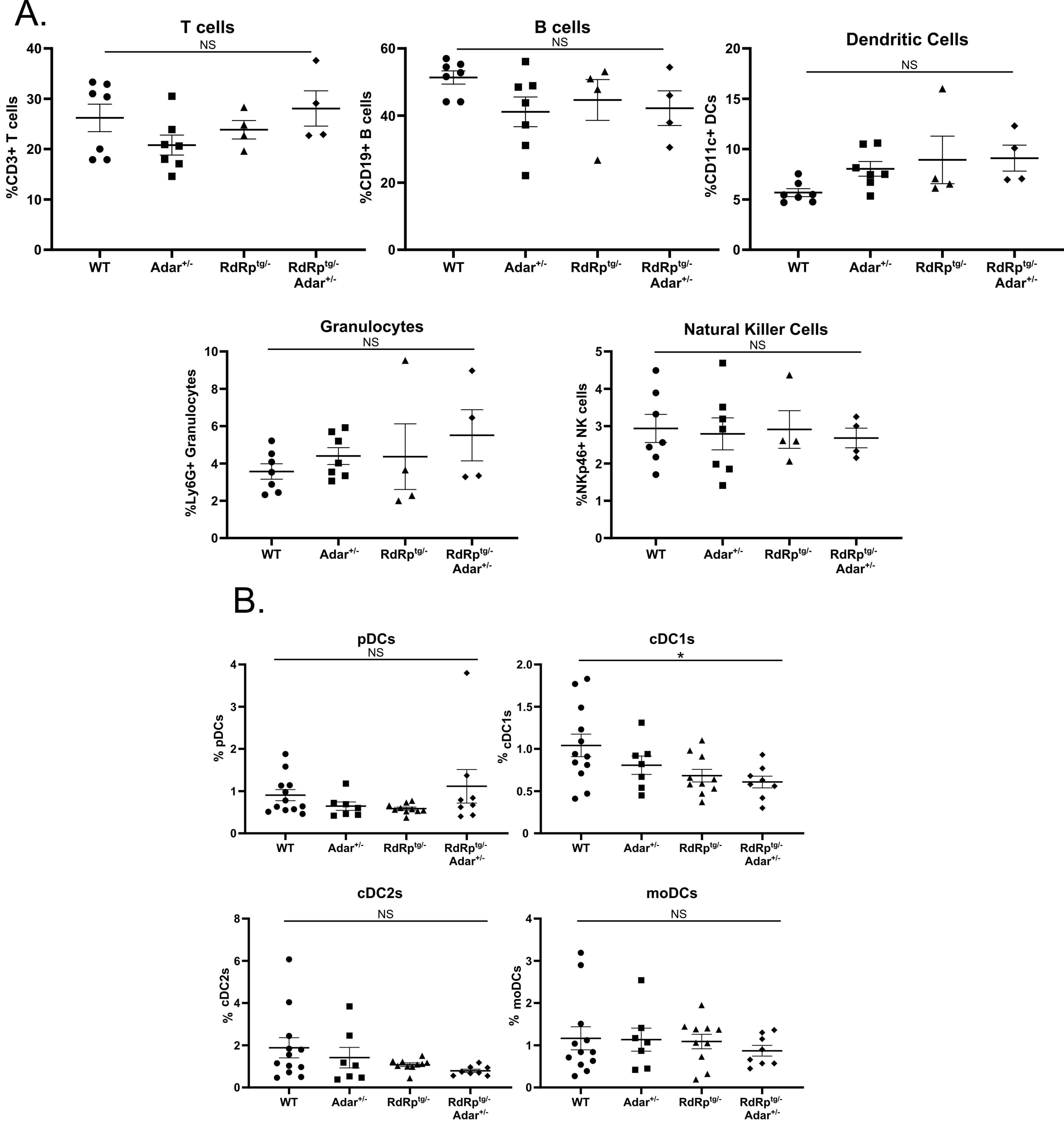


Supplemental Figure 7

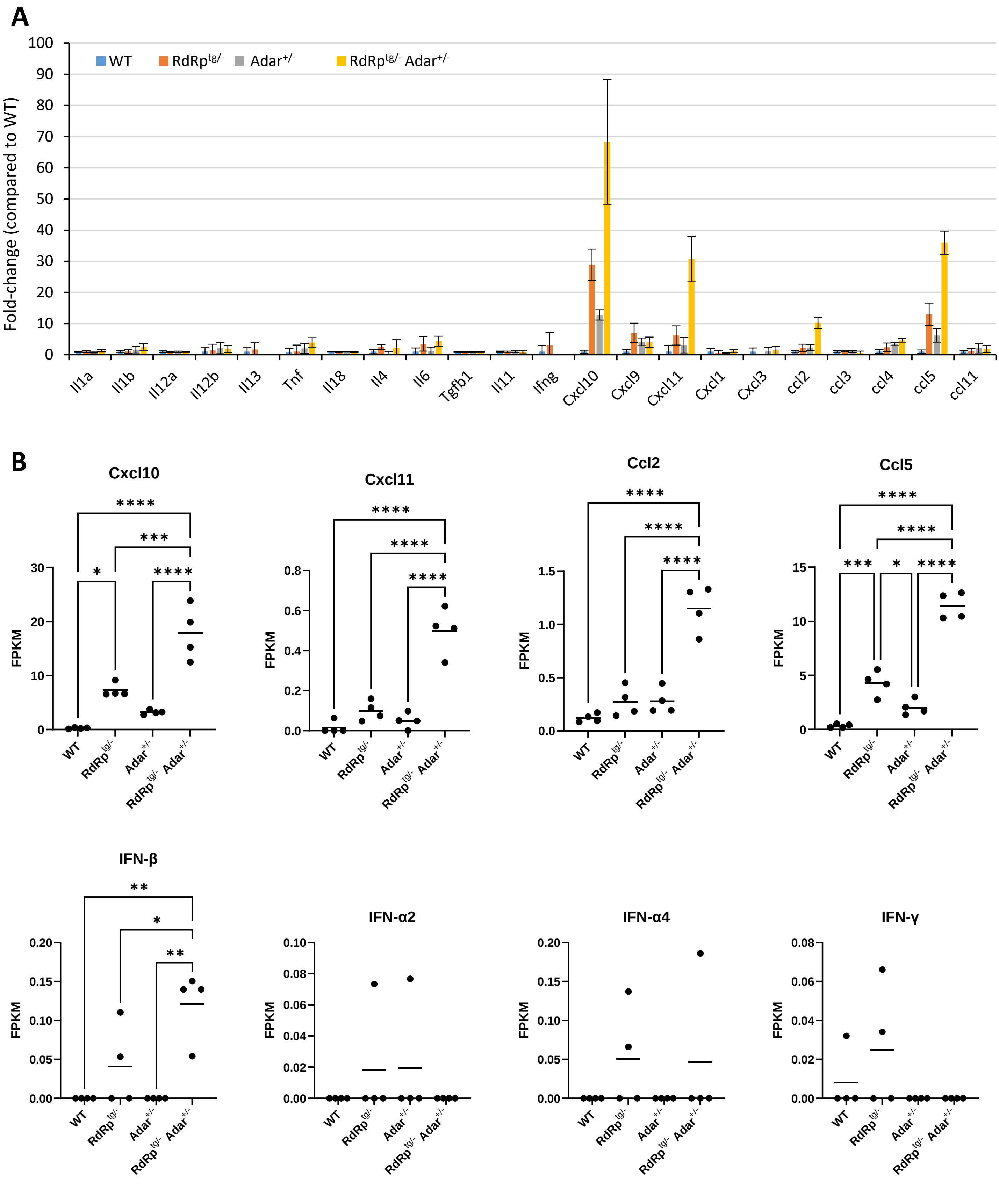


Supplemental Figure 8

| | WT vs. RdRp$^{tg/-}$ | | WT vs. Adar$^{+/-}$ | | WT vs. RdRp$^{tg/-}$ Adar$^{+/-}$ | | RdRp$^{tg/-}$ vs. Adar$^{+/-}$ | | RdRp$^{tg/-}$ vs. Adar$^{+/-}$ RdRp$^{tg/-}$ | | Adar$^{+/-}$ vs. Adar$^{+/-}$ RdRp$^{tg/-}$ | |
|---|---|---|---|---|---|---|---|---|---|---|---|---|
| **Diseases or Functions Annotation** | **-log(B-H p-value)** | **z-score** | **-log(B-H p-value)** | **z-score** | **-log(B-H p-value)** | **z-score** | **-log(B-H p-value)** | **z-score** | **-log(B-H p-value)** | **z-score** | **-log(B-H p-value)** | **z-score** |
| Antimicrobial response | 0.00000 | **2.39** | 1.97E-09 | 1.455 | NaN | NaN | 0.00000 | **-2.39** | 1.08E-05 | NaN | 1.21E-12 | 0.995 |
| Antiviral response | 0.00000 | **2.39** | 2.77E-11 | 1.455 | NaN | NaN | 0.00000 | **-2.39** | 2.82E-06 | NaN | 6.75E-12 | 0.995 |
| Apoptosis of breast cancer cell lines | NaN | NaN | NaN | NaN | NaN | NaN | NaN | NaN | NaN | NaN | 0.00146 | **2.808** |
| Apoptosis of epithelial cells | NaN | NaN | NaN | NaN | NaN | NaN | NaN | NaN | NaN | NaN | 0.00003 | **2.55** |
| Apoptosis of liver cells | NaN | NaN | NaN | NaN | NaN | NaN | NaN | NaN | NaN | NaN | 0.00146 | **2.169** |
| Cell death of epithelial cells | 0.00251 | **2.414** | NaN | NaN | NaN | NaN | NaN | NaN | 1.06E-03 | 0.922 | 1.41E-05 | **3.058** |
| Demyelination of nerves | NaN | NaN | NaN | NaN | NaN | NaN | NaN | NaN | 0.00001 | **2.195** | NaN | NaN |
| Differentiation of Th17 cells | NaN | NaN | NaN | NaN | NaN | NaN | | | NaN | NaN | 0.00014 | **-2.382** |
| Encephalitis | 3.09E-04 | -1.925 | 3.72E-07 | 0.264 | NaN | NaN | NaN | NaN | 1.09E-04 | -1.361 | 0.00000 | **-3.488** |
| Experimental autoimmune encephalomyelitis | 5.09E-04 | -1.639 | 7.57E-07 | 0.659 | NaN | NaN | 1.45E-06 | 1.881 | 1.66E-04 | -1.37 | 0.00000 | **-3.326** |
| Inflammation of absolute anatomical region | 8.46E-06 | -1.905 | 5.51E-08 | -0.527 | NaN | NaN | 1.39E-07 | 1.365 | 0.00002 | **-2.537** | 0.00000 | **-3.351** |
| Inflammation of organ | 3.08E-05 | -1.264 | 1.75E-07 | 0.255 | NaN | NaN | 1.71E-07 | 1.078 | 0.00001 | **-2.346** | 0.00000 | **-2.881** |
| Inhibition of cells | NaN | NaN | NaN | NaN | NaN | NaN | NaN | NaN | NaN | NaN | 0.00142 | **2.166** |
| Maturation of phagocytes | NaN | NaN | 2.29E-03 | -0.169 | NaN | NaN | 0.00074 | **-2.183** | NaN | NaN | 5.47E-04 | 0.891 |
| Necrosis | 8.48E-03 | 1.117 | NaN | NaN | NaN | NaN | NaN | NaN | 0.00160 | **2.156** | 0.00004 | **2.291** |
| Necrosis of epithelial tissue | NaN | NaN | NaN | NaN | NaN | NaN | NaN | NaN | 1.36E-04 | 1.014 | 0.00000 | **2.072** |
| Neuronal cell death | NaN | NaN | NaN | NaN | NaN | NaN | NaN | NaN | 0.00476 | **2.302** | 0.00006 | **2.309** |
| Proliferation of antigen presenting cells | NaN | NaN | NaN | NaN | NaN | NaN | 2.26E-03 | -0.865 | 3.64E-03 | NaN | 0.00014 | **2.201** |
| Proliferation of blood cells | 0.00421 | **2.084** | NaN | NaN | NaN | NaN | NaN | NaN | 9.98E-04 | 0.317 | 8.85E-06 | 0.535 |
| Proliferation of immune cells | 0.00429 | **2.084** | NaN | NaN | NaN | NaN | NaN | NaN | 9.14E-04 | 0.358 | 1.70E-05 | 0.808 |
| Quantity of blood cells | 0.00064 | **2.383** | NaN | NaN | NaN | NaN | 0.00193 | **-2.021** | 1.63E-04 | 0.487 | 2.79E-06 | 0.67 |
| Quantity of IL-12 in blood | 0.00002 | **-2** | 6.62E-03 | NaN | NaN | NaN | 0.00002 | **2** | NaN | NaN | 0.00007 | **-2** |
| Quantity of interleukin | NaN | NaN | NaN | NaN | NaN | NaN | NaN | NaN | NaN | NaN | 0.00064 | **-2** |
| Quantity of leukocytes | 0.00046 | **2.295** | NaN | NaN | NaN | NaN | 1.60E-03 | -1.906 | 1.02E-04 | 0.167 | 9.96E-07 | 0.336 |
| Quantity of phagocytes | 0.00104 | **2.191** | NaN | NaN | NaN | NaN | 2.21E-03 | -1.544 | 2.25E-05 | -0.125 | 5.80E-05 | -0.102 |
| Quantity of protein in blood | 0.00769 | **-2.909** | NaN | NaN | NaN | NaN | 0.00034 | **2.088** | NaN | NaN | 6.48E-04 | -1.717 |
| Rheumatic Disease | 0.00000 | **-2.219** | 7.94E-05 | -1 | NaN | NaN | 7.74E-07 | 1.179 | 3.07E-05 | -0.503 | 2.99E-08 | -1.157 |

Supplemental Figure 9

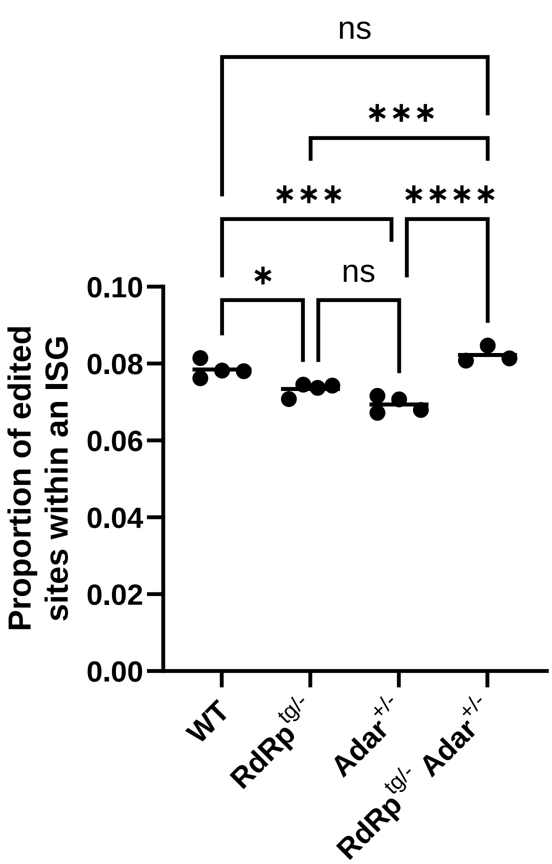


Supplemental Figure 10

## Supplemental Figure Legends

**Fig. S1. Overall B cell and T cell populations are marginally changed during BM12 transfer.** Single cell, RBC-lysed splenocyte suspensions were made from sham or BM12 transferred animals for flow cytometry analysis. **(A)** Overall B cell populations in animals as defined by cd19 expression. **(B)** Overall $CD4^+$ T cell population in the spleens of experimental animals, as defined by TCRβ and CD4 expression. Data were analyzed using a one-way ANOVA followed by a Tukey tests where * = $p < 0.05$, ** = $p < 0.01$, *** = $p < 0.001$, **** = $p < 0.0001$. n = 7, 6, 8, 8 for WT (PBS), *RdRp*$^{tg/-}$ (PBS), WT (BM12), and *RdRp*$^{tg/-}$ (BM12), respectively. Data represent individual animals with the mean and s.d. shown as bars.

**Fig. S2. Analysis of organ calcification.** Flash frozen brain and formalin-fixed paraffin embedded heart were collected from 6-week-old mice. **(A)** Heart samples were stained with Alizarin Red and calcium depositions were manually counted and shown as Alizarin Red foci per animal (n = 3, 5, 5, 10 for WT, *Adar*$^{+/-}$, *RdRp*$^{tg/}$, *RdRp*$^{tg/-}$ *Adar*$^{+/-}$, respectively). Representative heart section images are shown to the right: (i) positive control heart provided by UCDSOM Research Histology Core Laboratory; (ii) RdRp$^{tg/-}$ Adar$^{+/-}$ heart without Alizarin red foci; (iii) RdRp$^{tg/-}$ Adar$^{+/-}$ heart with a positive focus. **(B)** Calcium was quantified from brain tissues (n = 3, 4, 5, 5). Data were analyzed using a one-way ANOVA followed by a Tukey tests; there were no significant differences between the groups. ns: not significant.

**Fig. S3. Heatmap of differentially expressed ISGs across the four genotypes.** Enlarged image of Fig. 5B with gene names displayed. FPKM values were log transformed with 1 pseudocount to facilitate visualization.

**Fig. S4. Predicted upstream regulators.** Predicted upstream signaling regulators as determined by IPA in various genotype comparisons between WT, *Adar*$^{+/-}$, *RdRp*$^{tg/-}$*,* and *Adar*$^{+/-}$ *RdRp*$^{tg/-}$ mice where red/green indicates whether the gene itself is upregulated/downregulated in the RNA-seq and orange/blue indicates predicated activation/inhibition of the molecular pathway it regulates.

**Fig. S5. Loss of MDA5 rescues runting phenotype and abolished ISG upregulation.** 6-week-old animals were weighed **(A)** and RNA was isolated from whole-brains for qPCR analysis of the ISGs Ifit1 **(B)**, Isg15 **(C)**, and Oasl2 **(D)**. Data were analyzed using a one-way ANOVA followed by a Tukey tests where * = $p < 0.05$, ** = $p < 0.01$, *** = $p < 0.001$, **** = $p < 0.0001$. For (A), n = 4, 5, 6, 10, and 4 for WT, *Adar*$^{+/-}$, *RdRp*$^{tg/}$, *RdRp*$^{tg/-}$ *Adar*$^{+/-}$, and *RdRp*$^{tg/-}$ *Adar*$^{+/-}$ *Ifih1*$^{-/-}$, respectively. For (B-D), n = 4, 3, 5, 5, and 4. Data points represent individual animals with the mean for each group shown as a bar.

**Fig. S6. Disease and Biological Functions.** Enriched diseases and biological functions across comparisons of gene expression changed between WT, *Adar*$^{+/-}$, *RdRp*$^{tg/-}$*,* and *Adar*$^{+/-}$ *RdRp*$^{tg/-}$ mice. Orange indicated that the disease or biological function is predicted to be enriched for and blue indicated is predicted to be inhibited.

**Fig. S7. Certain immune cell populations are unchanged in *RdRp*$^{tg/-}$ *Adar*$^{+/-}$ mice.** Splenocytes from 4-5 week old mice were harvested and single cell, RBC-lysed solutions were made for FACS analysis. **(A)** T cells, B cells, DCs, granulocytes, and NK cells show no difference across groups measured. n = 7, 7, 4, and 4 for WT, *Adar*$^{+/-}$, *RdRp*$^{tg/}$, and *RdRp*$^{tg/-}$ *Adar*$^{+/-}$, respectively. **(B)** DC subsets including pDCs, cDC1s, cDC2s, and moDCs show minor or no difference in populations across groups. n = 12, 7, 10, and 8 for WT, *Adar*$^{+/-}$, *RdRp*$^{tg/}$, and *RdRp*$^{tg/-}$ *Adar*$^{+/-}$, respectively. ll data was analyzed using a one-way ANOVA followed by a Tukey

test to determine significance. * = $p < 0.05$. Data represent individual animals, graphs show means and s.d.

**Fig. S8. Chemokine, cytokine and IFN expression changes. (A)** RNA-seq-derived gene expression changes were determined for chemokines and cytokines and are shown as the average fold-change compared to wild-type mice, +/- s.d. **(B)** Individual animal FPKM values for cxcl10, cxcl11, ccl2, ccl5, IFN-β, IFN-α2, IFN-α4, and IFN-γ were graphed separately. Data were analyzed using a one-way ANOVA followed by a Tukey test to determine significance. No significant changes were observed for IFN-α2, IFN-α4, and IFN-γ. * = $p < 0.05$, ** = $p < 0.01$, *** = $p < 0.001$, **** = $p < 0.0001$.

**Fig. S9. Diseases and Biological Functions enriched for in A-to-I edited genes.** Genes with A-to-I edit sites and RNA-seq-derived expression changes were analyzed using IPA to determine enriched diseases and biological functions across genotype comparisons (WT, $Adar^{+/-}$, $RdRp^{tg/-}$, and $Adar^{+/-}$ $RdRp^{tg/-}$). Orange indicates that the disease or biological function is predicted to be enriched for or activated and blue indicates is predicted to be inhibited.

**Fig. S10. Proportion of edited sites in ISG RNAs as a fraction of all uniquely edited sites per animal**. Sites of A-to-I editing that were within an ISG were identified and the proportion of ISG edited sites out of all unique edited sites per animal was calculated. Bars indicate group means, dots represent individual animals, and the error bars are s.d. Data were analyzed using a one-way ANOVA followed by a Tukey tests where * = $p < 0.05$, *** = $p < 0.001$, **** = $p < 0.0001$, ns = not significant.

## Supplemental Methods, Miller et al.

### PCR primers and antibodies

**a. Primers**
***Mus musculus***
Gapdh: 5’-CATGGCCTTCCGTGTTCCTA-3’ & 5’-CTATGTTTCCCGCGGCACGTCAGATCCA-3’
Ifit1: 5’-TTTGTTGTTGTTGTTGTTCGTT-3’ & 5’-GCAGGAATCAGTTGTGATCT-3’
ISG15: 5’- TGGTACAGAACTGCAGCGAG -3’ & 5’- CAGCCAGAACTGGTCTTCGT -3’
Oasl2: 5’-CAAACAAACAAACAAACCCTCTC-3’ & 5’-TCAAGGTGTCACTCTGCAT-3’
Adar p150 5’-GGCCTTGCCGGCACTATGTCTC-3’ & 5’ GCGGGTATCTCCACTTGCTATGCTC-3’
Adar p110: 5’-GGTGGAAGACTACGCGTTGGGAC-3’ & 5’ ACGACTGTGTCTGGTGAGGGAACAC-3’
IFNβ: 5’-CACAGCCCTCTCCATCAACTATAAGCAG-3’& 5’-CTGTAGGTGAGGTTGATCTTTCCATTCAG-3’
***Homo sapiens***
GAPDH: 5’-TGGACCTGACCTGCCGT-3’ & 5’-TGGAGGAGTGGGTGTCGC-3’
ISG15: 5’-GTGGACAAATGCGACGAACC-3’ & 5’-ATTTCCGGCCCTTGATCCTG-3’
OASL: 5’-GGATCTTCTCCCACACTCACATCT-3’ & 5’-CACCATCAGGATTCTTCACGAA-3’

**b. Antibodies**
Rabbit polyclonal anti-ADAR1 (1:1,000 dilution; Cell Signaling, 14175)
Mouse anti-tubulin (1:5,000 dilution; Sigma, T5168)
Rabbit anti-Rig-I: mouse tissue blots (1:1,000 dilution, Cell Signaling, 3743)
Mouse anti-ADAR1 (1:500 dilution, Santa Cruz Bio, sc-73408)
Rabbit anti-actin (1:5,000 dilution, Cell Signaling, 4967)
Goat anti-rabbit secondary antibody, horseradish peroxidase -conjugated (1:5,000 dilution; Calbiochem, 401393)
Goat anti-mouse secondary antibody horseradish peroxidase-conjugated (1:5,000 dilution; Invitrogen, 31430)
Rabbit anti-ZBP1/DAI (1:1,000 dilution; Novus Biologicals NBP1-76854)
Rabbit anti-ISG15 (1:1000, Cell Signaling Technologies Cat # 2743)

**Quantitative PCR.** For qPCR analysis of mouse samples, RNA was extracted from mouse tissue using Trizol reagent (Ambion) according to manufacturer instructions. Reverse transcription PCR (RT-PCR) for cDNA synthesis was conducted using Maxima H Minus First Strand cDNA Synthesis Kit (Thermo Scientific) following manufacturer instructions. QPCR analysis for quantification of cellular transcripts was done using 2X qPCR Green Master Mix (APEX). For qPCR analysis from human cell lines, RNA extractions were done using an RNAeasy kit (Qiagen) according to kit protocol. RT- and qPCR were performed as described above. Transcripts were quantified using the $\Delta\Delta C_T$ method relative to *Gapdh* or β-Actin (*Actb*) transcripts.
**Immunoblotting.** Cells were homogenized in RIPA buffer and protein content was determined by Bradford reaction (Bio-Rad). 30 μg total protein was loaded per well prepared in 6X reducing SDS sample buffer (Bio-Rad, boil for 5-10 minutes prior to loading). Samples were run on 10% agarose gels (Bio-Rad) and transferred to polyvinylidene difluoride membrane (Bio-Rad). Samples were blocked in 5% milk in tris-buffered saline containing 0.1% Tween20 (TBS-T). Immobilon Crescendo Western HRP substrate (Millipore) was used for visualization of bands and imaged on a ChemiDoc XRS+ Imaging System (Bio-Rad).
**Cell culture and siRNA delivery**. Triplicate biological replicates were used with three technical replicates each. A549 cell lines (ATTC, CCL-185) were maintained in Dulbecco’s Modified

Eagle's Medium (Corning) containing 10% fetal bovine serum and penicillin/streptomycin/L-glutamine (Corning, 100x solution). Doxycycline-inducible RdRp-expressing cells were described previously (1). Two different siRNAs were used in combination for *ADAR* knockdown and were purchased from ThermoFisher Scientific (Catalog # 4390824, IDs s1008, s1009, previously validated). Negative control (ThermoFisher) or anti-*ADAR* siRNA were transfected into cells (50 pmol per well in a 6-well plate, cells seeded at >50% confluence) using lipofectamine RNAimax reagent (ThermoFisher) according to manufacturer instructions. Cells were washed 6 hours post transfection and dox (2 μg/mL) was added at this time. At 48 hours post dox addition, cells were lysed for protein and RNA analysis.

**Bioinformatics and Computational Methods**. We used a combination of RNA sequencing and exome sequencing to evaluate transcriptome-wide patterns of RNA gene expression and ADAR-mediated A-to-I RNA editing. Specifically, we sequenced four biological replicates from each of the four genotypes for RNA-seq (WT-*Adar*$^{+/+}$, WT-*Adar*$^{+/-}$, *RdRp*$^{tg/-}$ *Adar*$^{+/+}$, and *RdRp*$^{tg/-}$*Adar*$^{+/-}$) and a single sample from each genotype for exome sequencing. Exome sequencing was used to establish a baseline level of germline genetic variation within our mouse colony and was used as background for identifying RNA-edited sites. Library preparation and sequencing for single-end RNA-sequencing and paired-end exome sequencing were performed by BGI (Beijing Genomics Institute). The quality of raw reads was assessed using FASTQC v.0.11.5. We used Trimmomatic v.0.27 to filter out low quality reads that had a mean Phred quality score less than 20. Read quality was then reassessed with FASTQC. We used Hisat2 v.2.1.0 to map reads to the mm10 genome using default single-end parameters for RNA-seq and included the --no-mixed --no-discordant flags for the paired-end exome sequencing. Next we used Samtools v.1.3.1 to convert the sam output files into bam format. Then we used PicardTools v.1.119 to add read groups, remove PCR duplicates, and coordinate sort the bam files. To evaluate RNA-seq gene expression patterns across the four genotypes, we first used Stringtie v.1.3.6 to quantify transcripts abundance, and Stringmerge to merge the output Stringtie GTF files. CuffDiff2 was to identify significantly differentially expressed genes (DEGs) with an FDR of 0.05 and Benjamini-Hochberg multiple test correction. In order to reduce false positives, we also required fragments per kilobase per million reads mapped (FPKM) to be at least four, and at 2-fold differences in expression because of the massive gene expression differences observed in(1, 2). We used CummeRbund and Plotly to visualize our results. The Interferome Database was used to identify which DEGs were known ISGs. Finally, we used the Canonical Pathways and Diseases and Biological Functions analyses in IPA to determine which molecular pathways and biological functions were enriched among our conditions and to predict whether they were likely to be activated or inhibited based on gene expression patterns in our dataset. To identify RNA-edited sites, we used REDItools to call SNPs that were present in the RNA-seq, but not present in the exome sequencing. Because the A-to-I editing performed by ADAR is incorporated as A-to-G changes by sequencers, we looked for differences in A-to-G changes across conditions. We required a minimum of 10x coverage and that each putative RNA-editing SNP be sequenced at least twice to reduce the risk of including sequencing errors. First, we used Samtools mpileup to call SNPs in the exome sequencing data. We used REDItoolsDeNovo to call SNPs A-to-I edited sites in the RNA-seq requiring at least 10x coverage and that each SNP was sequenced at least twice. Putative germline SNPs identified in the exome sequencing were subtracted from the RNA SNPs. The resulting SNP tables were converted into bed format and sorted by coordinates using BedTools and we filtered SNPs to those present in all four biological replicates. We then annotated the SNPs using Homer annotatePeaks.pl to determine the genomic features in which SNPs occur. A-to-I editing patterns were compared across the four genotypes in two ways and visualized using SUMO, 1) whether editing occurred in the same vs. different genomic features, allowing editing sites to occur at different locations within that feature, and 2) whether editing occurred at identical sites. Finally, we used IPA to identify significantly enriched molecular pathways and biological functions associated with edited genes

among the 4 genotypes. The RNA-seq and whole exome sequencing datasets have been deposited to the NCBI sequence read archive (SRA SUB14698103).

**Flow Cytometry**. Mice were euthanized by $CO_2$ asphyxiation and subsequent cardiac puncture. Spleens were excised and placed in 15 mL conical tubes containing 3 mL of DMEM containing FBS and antibiotics. Spleen was manually dissociated with a pipette tip and pipetting, then passed through a 40 μM nylon cell strainer. 100 μL from each sample were combined and used for the isotype and unstained controls. Cells were gated to exclude debris, doublets, and dead cells based on viability dye staining. Cell population abundance: samples had at least 1x10^6 cells sorted in the live population and cell types are shown as percent of live cells for all flow cytometry data. Each cell type described had a minimum of about 0.5% meaning all cell types described were observed at a frequency of about 5,000 or more per sample. Analysis of cellular populations was conducted as previously described (3). For staining of splenocyte populations, cells were red blood cell depleted as described (3). For analysis including FoxP3 staining, intracellular transcription factor staining was done using Foxp3/Transcription Factor Staining Buffer Set (eBioscience) according to manufacturer instructions for use in a 96-well plate. DC-specific staining panel has been previously described (3). $T_{reg}$ staining panel consists of the following antibodies/cellular stains: fixable viability dye eFlour780 (eBioscience, 65-0865-14), PE anti-mouse FoxP3 (BioLegend, 126404), PerCP-Cy5.5 anti-mouse CD4 (BioLegend, 100434), Brilliant Violet 421 anti-mouse CD25 (BioLegend, 101923), APC anti-mouse CTLA-4 (BioLegend, 106310), Brilliant Violet 711 anti-mouse GITR (BD Bioscience, 563390), Brilliant Violet 605 anti-mouse ICOS (BioLegend, 313538), Brilliant Violet 480 anti-mouse CD44 (BD Bioscience, 566116) and FITC anti-mouse CD62L (BioLegend, 104406). For analysis of B cells (germinal center and plasmablast) the following antibodies and cellular stains were used: fixable viability dye eFlour780 (eBioscience, 65-0865-14), Brilliant Violet 421 anti-mouse CD138 (BioLegend, 142507), PE anti-mouse Fas (BD Bioscience, 554258), Alexa Fluor 488 anti-mouse GL-7 (BioLegend, 144612). For $T_{FH}$ cell analysis and CD45.1 engraftment, the following antibodies and cellular stains were used: fixable viability dye eFlour780 (eBioscience, 65-0865-14), PerCP-Cy5.5 anti-mouse CD4 (BioLegend, 100434), Brilliant Violett 711 anti-mouse TCRβ (BioLegend, 109243), PE anti-mouse PD-1 (BioLegend, 135205), APC anti-mouse CXCR5 (BioLegend, 145506), and Brilliant Violet 421 anti-mouse CD45.1 (BioLegend, 110732). BST-2 was detected with Pacific Blue anti-mouse BST-2 (BioLegend, 127108). Gating for B cell and $T_{FH}$ cell analysis was described in ref (4). Isotype staining for each panel was done in parallel. Gating was set based on fluorescence minus one controls. Cells were gated to exclude debris, doublets, and dead cells based on viability dye staining. Gating for DC-subtype determination was described in ref. (3). All data was acquired on an LSRII (BD Biosciences) and analysis was done using FlowJo software.

**ELISAs**. Mouse serum was used for ANA detection via ELISA. For detection of anti-dsDNA antibodies, calf thymus DNA (Sigma, D4522) was digested with S1 nuclease (ThermoFisher, EN0321) to remove any single stranded DNA (ssDNA). DNA was ethanol precipitated and resuspended in nuclease free water. Freeze-thaw cycles were avoided to prevent creation of new ssDNA. Nunc MaxiSorp ELISA plates (Sigma) were coated with Poly-L Lysine (0.01% final, Millipore Sigma) prior to use. Plates were coated overnight with dsDNA (3 μg/mL) in PBS at room temperature and blocked with BSA in PBS (3% final, Sigma). For a standard, anti-dsDNA (Abcam, ab27156) was used between the range of 1 μg/mL – 1.37 ng/mL (3-fold dilutions) and serum samples were initially diluted 1:10 and then as needed. For anti-smAg ELISAs, plates were coated with Sm Ag (3 μg/mL, Genway Biotech, GWB-CA4A65) in PBS overnight. Blocking was done in 1% BSA/PBS. For standard, anti-SmAg (Novus Biologicals, NB600-546) was used between the range of 1 μg/mL – 15.63 ng/mL (2-fold dilutions) and serum samples were diluted 1:10 before use and then as needed. For both ELISAs, standard and serum samples were diluted in sample buffer (0.5% Tween20, 1% BSA in PBS) and plates were washed 3-5 times between incubations with wash solution (0.5% Tween20 in PBS). Plates were incubated with samples/standard for 2 hours at 37°C. Detection antibody used was goat anti-mouse IgG

peroxidase (1:10,000 dilution; Sigma, A2554) and peroxidase was developed used TMB ELISA HRP Substrate kit (Seracare) and Sulfuric acid (4N, Fisher Scientific) according to manufacturer instructions.

**Calcium Quantification.** Whole-brain tissue was flash frozen at necropsy, homogenized by sonication on ice in 4x volumes of calcium assay buffer (Calcium Assay Kit, Abcam ab102505), and free $Ca^{2+}$ ions were quantified following the manufacturer's methods.

**Histopathology**. Briefly, approximately 2 cm thick tissue sections of 5 week old mice were fixed in 10% formalin for 24 hours then washed twice and suspended in 70% ethanol. This age was chosen as by this point the mice already showed notable changes in growth but had not yet begun to succumb to disease related mortality. Tissues were paraffin embedded, cut and stained for Alizarin Red or H&E by our Research Histology Core Laboratory. H&E-stained slides were analyzed and scored by the IDEXX BioAnalytics histopathology group and are shown in the figure below. Grading was: 0 no change from WT, 1 minimal, 2 mild, 3 moderate, 4 marked changes. All scores were zero except for one RdRp$^{tg}$ kidney which was scored 1.

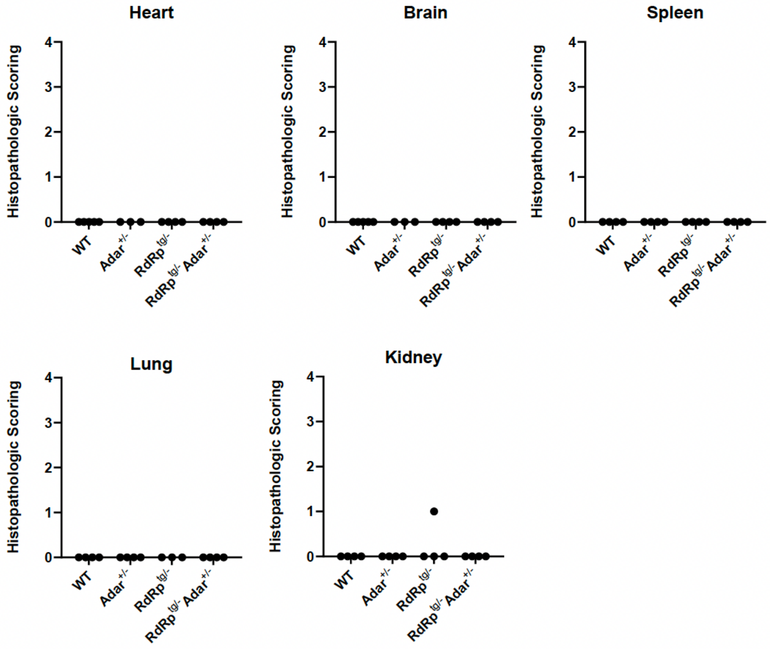


Figure. Histopathological grading of mouse tissues. No significant changes or differences were noted in the RdRp$^{tg/-}$ Adar$^{+/-}$ animals to explain the clinical presentations of this genotype.

References for Supplemental Methods


1. Painter MM, Morrison JH, Zoecklein LJ, Rinkoski TA, Watzlawik JO, Papke LM, et al. Antiviral Protection via RdRP-Mediated Stable Activation of Innate Immunity. *PLoS pathogens.* 2015;11(12):e1005311.
2. Bankers L, Miller C, Liu G, Thongkittidilok C, Morrison J, and Poeschla EM. Development of interferon-stimulated gene expression from embryogenesis through adulthood, with and without constitutive MDA5 pathway activation. *Journal of Immunology.* 2020;204(10):2791-807.
3. Miller CM, Barrett BS, Chen J, Morrison JH, Radomile C, Santiago ML, et al. Systemic Expression of a Viral RdRP Protects against Retrovirus Infection and Disease. *Journal of virology.* 2020;94(9).
4. Klarquist J, and Janssen EM. The bm12 Inducible Model of Systemic Lupus Erythematosus (SLE) in C57BL/6 Mice. *J Vis Exp.* 2015(105):e53319.
